# Video-Assisted Thoracoscopic Surgery (VATS) in Pregnant Patient With Tuberculosis: A Case Report

**DOI:** 10.7759/cureus.67933

**Published:** 2024-08-27

**Authors:** Dragan Rakanovic, Suzana Sobot Novakovic, Marko Kantar, Sanja Rakanovic, Ivana Popovic

**Affiliations:** 1 Clinic for Anesthesiology and Intensive Therapy, University Clinical Centre of the Republic of Srpska, Banja Luka, BIH; 2 Faculty of Medicine, University of Banja Luka, Banja Luka, BIH; 3 Clinic for Thoracic Surgery, University Clinical Centre of the Republic of Srpska, Banja Luka, BIH

**Keywords:** pneumonia, empyema, video-assisted thoracoscopic surgery (vats), tuberculosis, pregnancy

## Abstract

Diagnosis of tuberculosis (TB) in pregnancy may be challenging. Recommended diagnostic tests often are not sensitive, and additional diagnostic procedures are necessary to confirm disease. Symptoms of TB in pregnancy are often atypical and difficult to diagnose in the early stages of the disease.

Obstetric complications of TB include spontaneous abortion, preterm labor, low birth weight, and increased neonatal mortality. In pregnant patients, empyema is one of the complications of tuberculous pneumonia, and video-assisted thoracoscopic surgery (VATS) is the recommended surgical treatment.

We present the case of a pregnant patient in the 20th week of gestation who was hospitalized due to suspected TB. Serological, microbiological, and molecular tests specific to TB were negative. Radiological tests confirmed pneumonia with pleural effusion. Due to the development of empyema, VATS debridement was indicated. VATS pleural biopsy confirmed the diagnosis of TB.

## Introduction

Tuberculosis (TB) is the second leading infectious cause of mortality globally after COVID-19. In 2022, 1.3 million people died from TB; about two-thirds of the cases were found in China, the Philippines, Pakistan, Nigeria, Bangladesh, and the Democratic Republic of the Congo. Of 10.6 million new TB diagnosed each year, 3.5 million are in females [1]. Epidemiology data about TB in pregnancy are not routinely collected. In the absence of systematically collected data, global modeling studies estimate 200,000 incident TB diagnoses during pregnancy each year. It is estimated that 900 million women worldwide have latent *Mycobacterium tuberculosis* (MTB). Pregnant women with MTB are more likely to develop active TB compared to men [2]. Active TB disease during pregnancy remains associated with a substantially elevated risk for poor maternal and fetal outcomes, including a three-fold increase in maternal morbidity (e.g., antenatal admission, anemia, preeclampsia, and a cesarean delivery (CD)), a nine-fold increase in miscarriage, a two-fold increase in preterm delivery and low birth weight, and a six-fold increase in fetal and neonatal death [3]. Risk factors for the progression of TB infection include past TB infection, contact with infected individuals, immigration, poor health and malnutrition, drug abuse, alcoholism, smoking, living conditions, and immunosuppression [4]. In areas with a high incidence of disease, universal screening for TB among pregnant women is recommended. For regions with a low disease burden, screening should target selected at-risk pregnant patients for early case finding. The most common manifestation of TB in pregnancy is pneumonia [5]. Empyema is one of the complications of TB pneumonia in pregnancy [6]. The treatment of empyema depends upon the stage of the disease. Video-assisted thoracoscopic surgery (VATS) is the preferable surgical treatment in the management of stage II and III empyema [7]. VATS in pregnant patients is not that common, and only a few cases are described in the literature [8-11].

## Case presentation

A 23-year-old female patient in the 20th week of gestation was admitted to the hospital with symptoms of cough, chest pain, and fever in the seven days before admission. Her medical and surgical history was unremarkable, but her sister had active pulmonary TB a few years ago. Physical examination of her breath sounds revealed crackles in the right base. Laboratory tests show elevated C-reactive protein (CRP) and normal white blood count (WBC) with lymphopenia. Due to the suspicion of pneumonia, antibiotics were prescribed (ceftriaxone 2 × 2000 mg, 10 days).

**Figure 1:**
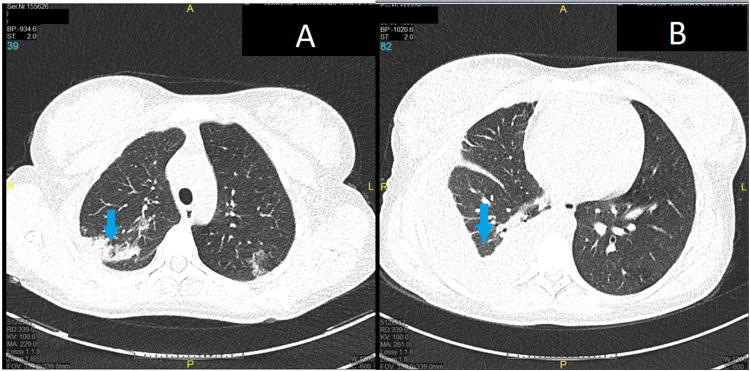
Low-dose CT of the lungs showing bilateral pulmonary consolidation (A) CT slide showing bilateral pulmonary consolidation predominantly on the right side (blue arrow). (B) CT slide showing pleural effusion (blue arrow).

**Figure 2:**
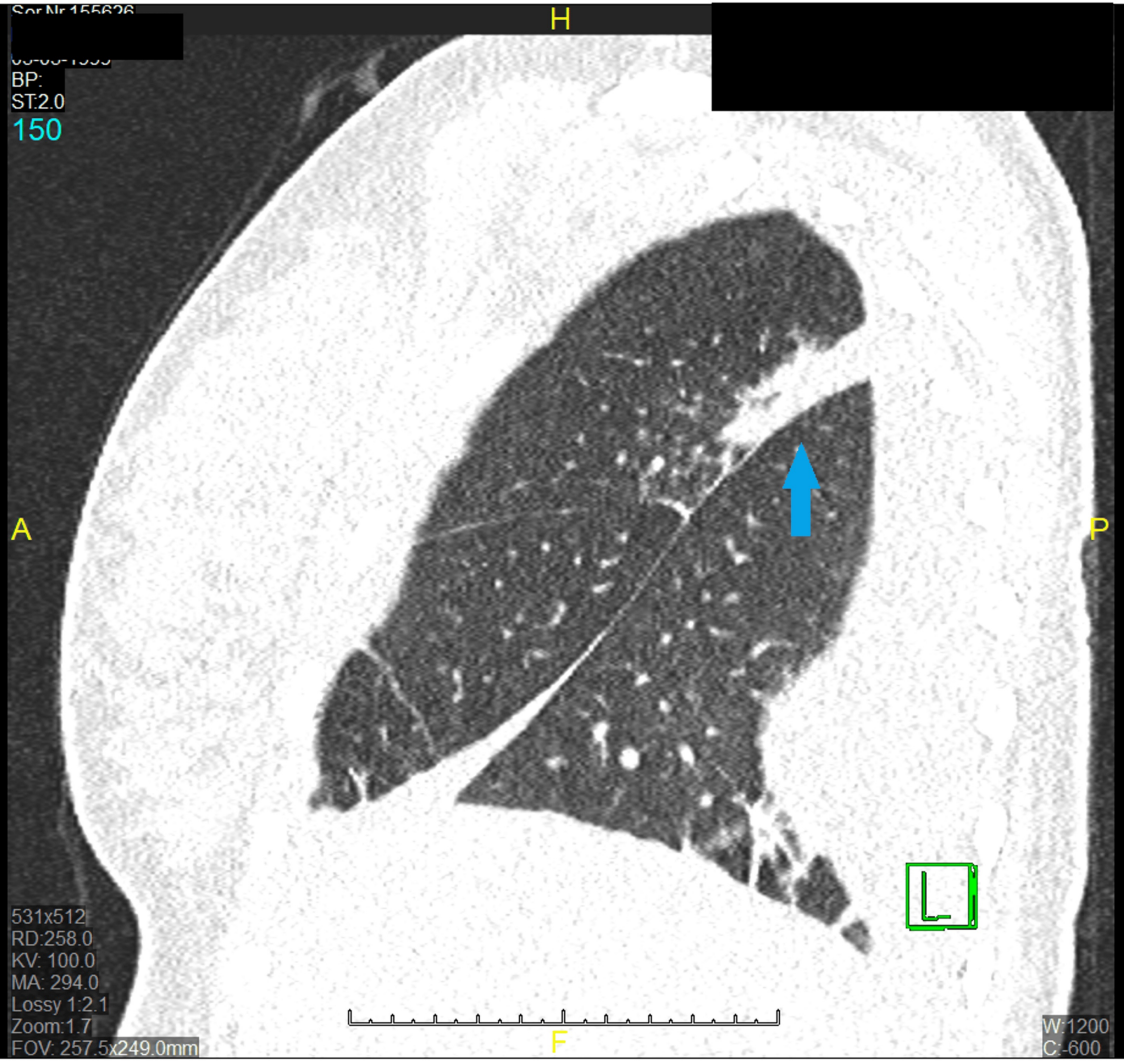
Low-dose CT of the lungs showing pulmonary consolidation A sagittal plane view of the CT scan showing pulmonary consolidation on the right side (blue arrow).

For the differential diagnosis of infections, the following tests were performed: urine and blood culture; sputum analysis; respiratory PCR panel for 22 pathogens, including SARS-CoV-2, influenza A and B virus, *Chlamydia pneumoniae*, and *Mycoplasma pneumoniae*; urine tests for *Legionella pneumophila* antigen and *Streptococcus pneumoniae* antigen; and serological tests for SARS-CoV-2 and cytomegalovirus, which were all negative. TB tests included the QuantiFERON test (interferon-gamma release assay (IGRA), blood test) and sputum analysis (MTB microscopic, Ziehl-Neelsen stain), which were negative. Laboratory tests for systemic autoimmune disease were also negative. A lung ultrasound detected pleural effusion on the right side. The multidisciplinary team decided to perform a chest X-ray, which showed right pulmonary consolidation located near the pericardium with pleural effusion reaching up to the third rib. After ultrasound-guided thoracentesis, the sample of pleural effusion was analyzed. Cytological analysis shows mixed cellularity with a predominance of lymphocytes; microbiological analysis shows no bacteria microscopically, and the culture remained sterile. Finally, the line probe assay (LPA) polymerase chain reaction (PCR) that was used to detect MTB was also negative. A low-dose CT scan of the lungs showed bilateral pulmonary consolidation with pleural effusion on the right side, which suspected atypical pneumonia (TB or fungal) (Figures 1, 2).

**Figure 3:**
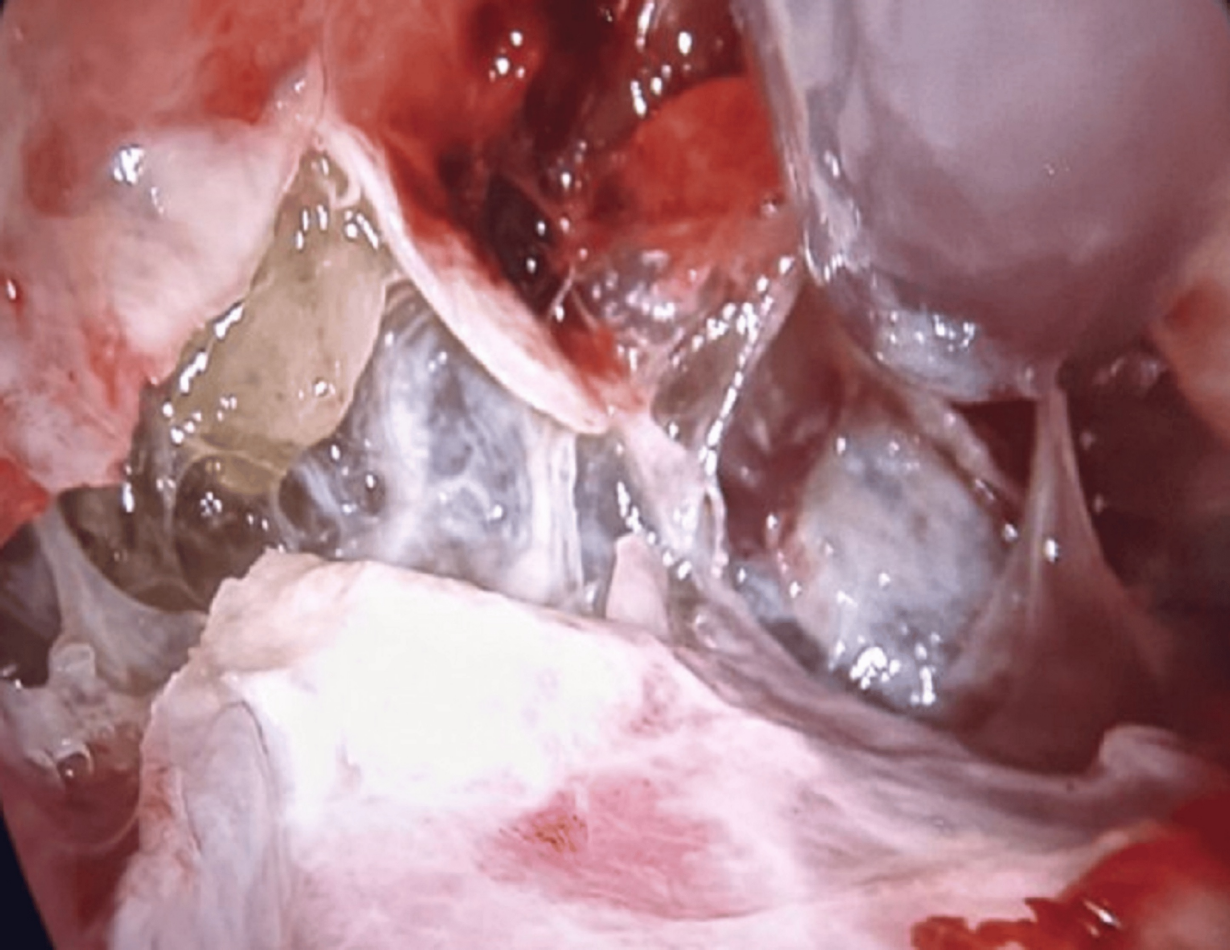
Liquid collection in the gel phase between visceral and parietal pleura

Antibiotic therapy was changed during hospitalization, and at that moment, the patient was treated with meropenem in a dose of 3 × 1000 mg on the ninth day of treatment and the 20th day of hospitalization. Inflammatory parameters were elevated from the beginning of the disease, such as CRP: 94, 84, 54, 82, and 21 mg/L and WBC: 9.14, 7.24, 6.6, and 8.6 × 10^9^. The patient had chest pain and was febrile throughout the course of the disease every day, with subfebrile temperature periods of 37.5°C and mild fever periods of 38.5°C. The highest temperature was at the beginning of the disease, and with the reduction of inflammatory parameters, the temperature also decreased. It was indicated that uniportal VATS should be performed for debridement and sampling of pleural effusion and parietal pleura for microbiological, biochemical, and histological tests. Perioperative management included a multidisciplinary approach and follow-up by the obstetrician, cardiologist, pulmonologist, and anesthesiologist. Preoperative fetal ultrasound showed a viable pregnancy. The patient’s American Society of Anesthesiologists (ASA) physical status was III. Prior to induction to anesthesia, the patient received 2 mg of midazolam and 150 µg of fentanyl. Intraoperative monitoring included invasive blood pressure (FloTrac Edwards Lifesciences EV1000), ECG monitoring, pulse oximetry, BIS monitoring, end-tidal CO2, and neuromuscular monitoring. After three minutes of preoxygenation with 100% oxygen, general anesthesia was induced with 150 mg propofol, followed by atracurium 50 mg. The patient was intubated with a left-side double-lumen endobronchial tube (DLT) size of 35 Fr (controlled with fiberoptic bronchoscope). Anesthesia was maintained with 50% oxygen, MAC 0.7 of sevoflurane, and 200 µg fentanyl. The patient was placed in the left lateral position. Right lung isolation and protective one-lung ventilation were performed with a tidal volume of 4-6 mL/kg, respiratory rate of 14-18 breaths per minute (according to end-tidal CO2), PEEP of 5 cmH2O, and peak pressure of 10-12 cmH2O. The total operating duration was 40 minutes. Intraoperative thoracoscopic images showed disseminated micronodules and liquid collection in the gel phase (Figure 3). The patient was extubated and postoperatively transferred to the intensive care unit (ICU). Postoperative fetal ultrasound was normal.

Biochemical analysis included a total protein of 46 g/L, LDH of 664 UI/L, and glucose level of 2.6 mmol/L, indicating an inflammatory process or malignancy (exudative effusion). Analysis of the parietal pleura sample included LPA PCR to detect MTB and culture of the parietal pleura sample, which were negative. Pathohistological findings of a biopsy of the parietal pleura confirmed a diagnosis of chronic granulomatous inflammation, i.e., TB. Tuberculostatic therapy (isoniazid, rifampicin, and ethambutol) was started. The patient was discharged home after 45 days of hospitalization in the 26th week of pregnancy, in overall good condition and viable pregnancy.

In the 30th week of gestation, the patient was readmitted to the hospital due to reduced fetal movements. An ultrasound revealed no fetal heartbeats. Labor was induced with medication (misoprostol and oxytocin) on the same day after the admission. The epidural catheter was placed (combined spinal epidural analgesia), and it was a stillborn female weighing 1560 g and length of 44 cm. Placental biopsy confirmed infarction of the placenta.

## Discussion

Pregnant women are at increased risk of TB during pregnancy because of the immunological changes associated with pregnancy. Diagnosis of TB in pregnancy is difficult due to the non-specific symptoms, the increased incidence of an extrapulmonary form of the disease, the delay in performing radiological diagnostic tests, and the high rate of tuberculin-negative tests. A review made by Kothari et al. shows that the median diagnostic delay for TB in pregnancy is 32 days [12]. The diagnosis of TB pneumonia is usually delayed because pregnant patients are being postponed to have chest radiography. Recommended diagnostic tests for TB are smear microscopy, culture, and molecular DNA detection methods, such as XpertMTB/RIF. Microscopy has a low sensitivity and cannot distinguish MTB from other mycobacterial organisms. Culture can take more than four weeks to yield a result. WHO recommended the replacement of microscopy with molecular rapid diagnostic tests [13]. A study among HIV-positive pregnant women in Kenya reported an XpertMTB/RIF sensitivity of 43% and a specificity of 100% when compared to MTB culture results as the gold standard [14].

In our case, the patient had clinical symptoms and a medical history of being in contact with a person diagnosed with TB. Chest X-ray and low-dose CT scan showed pulmonary consolidations with pleural effusion. Recommended diagnostic tests for TB diagnosis were negative. This delayed the initiation of tuberculostatic agent therapy. Pneumonia was complicated by empyema. VATS was indicated for debridement and pleural biopsy.

Minimally invasive surgery, such as VATS, enables fast recovery while minimizing complications. There are several published case reports of performing VATS in pregnancy. Oshodi et al. reported a pregnant patient in the 25th week of gestation where VATS was performed for the treatment of empyema [8]. The procedure was successful, without complications for the mother and the fetus [8]. In another case report, Kim et al. performed VATS on a 38-year-old patient in the 24th week of gestation for the treatment of lung cancer [9]. In this patient, pulmonary lobectomy was performed without any complications. Pregnancy ended in the delivery of a healthy boy [9]. Nwaejike et al. report a case of recurrent chest-tube-resistant pneumothorax during the third trimester of pregnancy; VATS was performed for bullectomy and pleurodesis, which was successful without either peri-operative or peripartum complications [10]. Hu et al. also reported a recurrent spontaneous pneumothorax in a twin pregnancy within a 30-year-old patient at the 22nd week of gestation [11]. VATS was successfully performed without complications [11]. According to the data presented in the literature, VATS is tolerated well by pregnant patients and fetuses. Special attention should be given to anesthesia management and supportive measures in the perioperative period.

TB is recognized as one of the most common infectious diseases causing morbidity and mortality during pregnancy. Active TB increases the risk of complications in pregnancy, including preterm delivery, spontaneous abortion, severe preeclampsia, stillbirth, intrauterine fetal growth restriction, and low birth weight [15]. Although TB is associated with adverse pregnancy outcomes, the pathogenesis is not fully understood. Different mechanisms have been described, such as direct microorganism effects on the fetus or placenta or effects mediated by pathogen-induced immune responses. The placental vascularization may be affected by maternal immune response, which in turn could have adverse effects on pregnancy outcomes [16]. Walles et al. found a significantly increased incidence of stillbirth, severe preeclampsia, emergency cesarean section, and low infant birth weight in pregnancies of women with TB [17]. Figueroa-Damian et al. reported substantially increased neonatal mortality as a result of late diagnosis and treatment [18].

## Conclusions

TB in pregnancy is a rare disease in our country, and screening tests are not routinely performed in pregnant women. The diagnosis of TB in pregnancy can be difficult. Non-specific symptoms and very often negative tests for the detection of MTB and TB can lead to delayed diagnosis and late initiation of therapy. This can lead to serious complications for the mother and fetus. VATS in pregnancy is a safe procedure, and there were no maternal or fetal complications reported from this procedure during pregnancy. VATS enables fast recovery while minimizing complications, and it can be recommended for all procedures in thoracic surgery during pregnancy if there is an experienced surgeon, anesthesia team, and technical conditions. Anesthesia management and adequate perioperative care are essential for preventing perioperative complications.
